# How Image-Guided Pathology Can Improve the Detection of Lymph Node Metastases in Prostate Cancer

**DOI:** 10.1097/RLU.0000000000004158

**Published:** 2022-04-19

**Authors:** Melline G.M. Schilham, Heidi Küsters-Vandevelde, Diederik M. Somford, M. Rijpkema, Martin Gotthardt

**Affiliations:** From the ∗ Department of Medical Imaging, Nuclear Medicine; † Department of Urology, Radboud University Medical Centre; Departments of ‡ Pathology; § Urology, Canisius Wilhelmina Hospital; ¶ Prosper Prostate Cancer Clinics, Nijmegen, the Netherlands.

**Keywords:** image-guided pathology, PSMA, lymph node, staging, prostate cancer

## Abstract

Detection of lymph node (LN) metastases in prostate cancer (PCa) is pivotal for accurate staging and determining treatment options. To date, the reference standard for nodal staging is histopathological examination of all harvested surgical specimens from extended pelvic LN dissections. However, this is a labor-intensive process, and small metastatic foci can be missed due to sampling effects. With current research expanding toward using radiolabeled prostate-specific membrane antigen ligands for image-guided surgery, new opportunities arise for image-guided pathological assessment of surgical specimens. Here, we illustrate how molecular imaging can complement histopathology and improve accurate detection of LN metastases.

**FIGURE 1:**
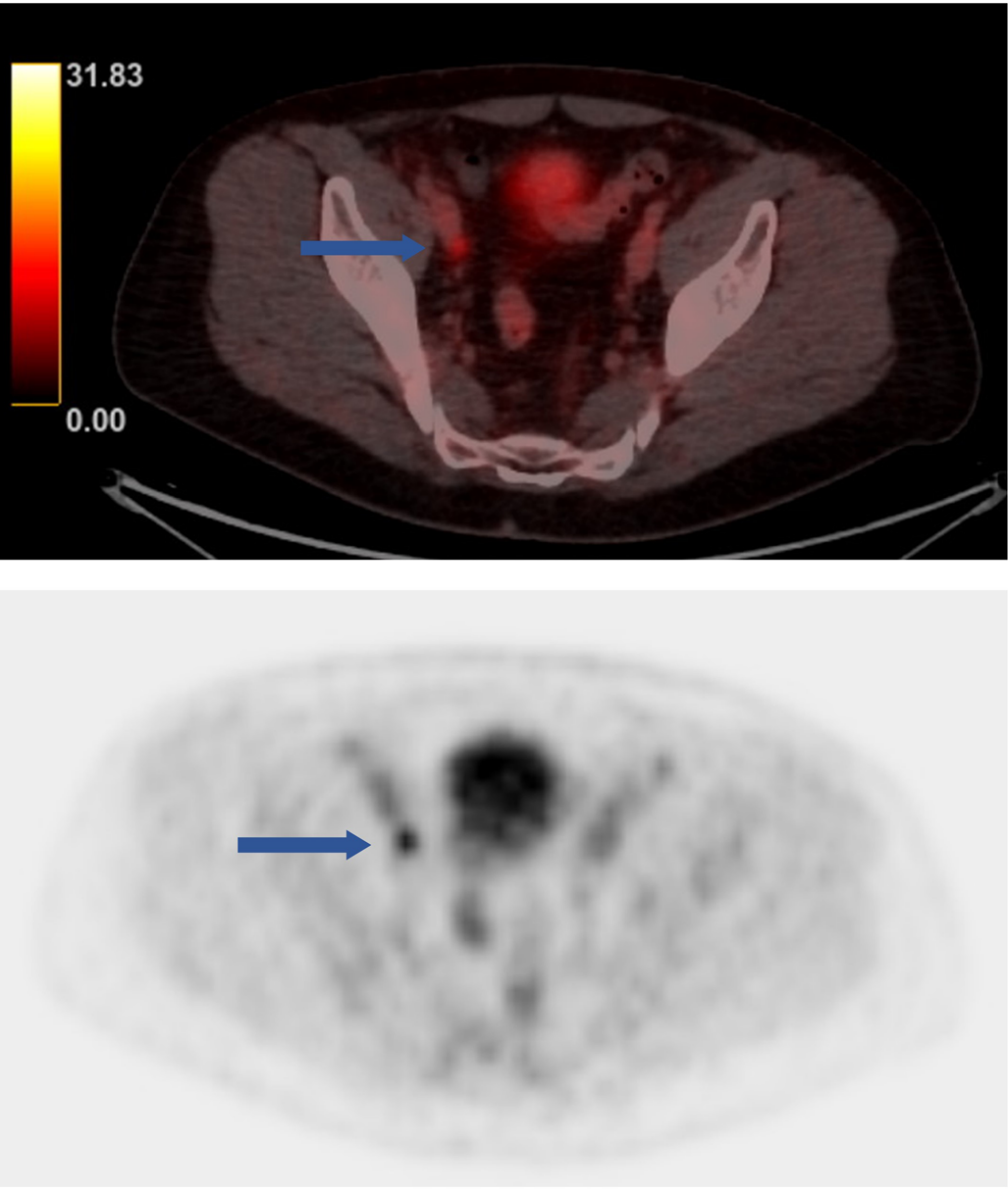
A 66-year-old man with a prostate-specific antigen level of 15 ng/mL was diagnosed with an adenocarcinoma of the prostate. According to international guidelines,^1^ a preoperative ^18^F-rhPSMA-PET/CT^2^ was performed and revealed 2 prostate-specific membrane antigen (PSMA)–avid lymph nodes (LNs), one in the right obturator region (blue arrow) and one in the presacral region (not shown). Radio-guided, robot-assisted extended pelvic LN dissection using a laparoscopic γ-probe was performed 22 hours after IV administration of ^111^In-PSMA-I&T^3^ (NCT04300673). Both LNs were successfully detected and removed in packets.

**FIGURE 2:**
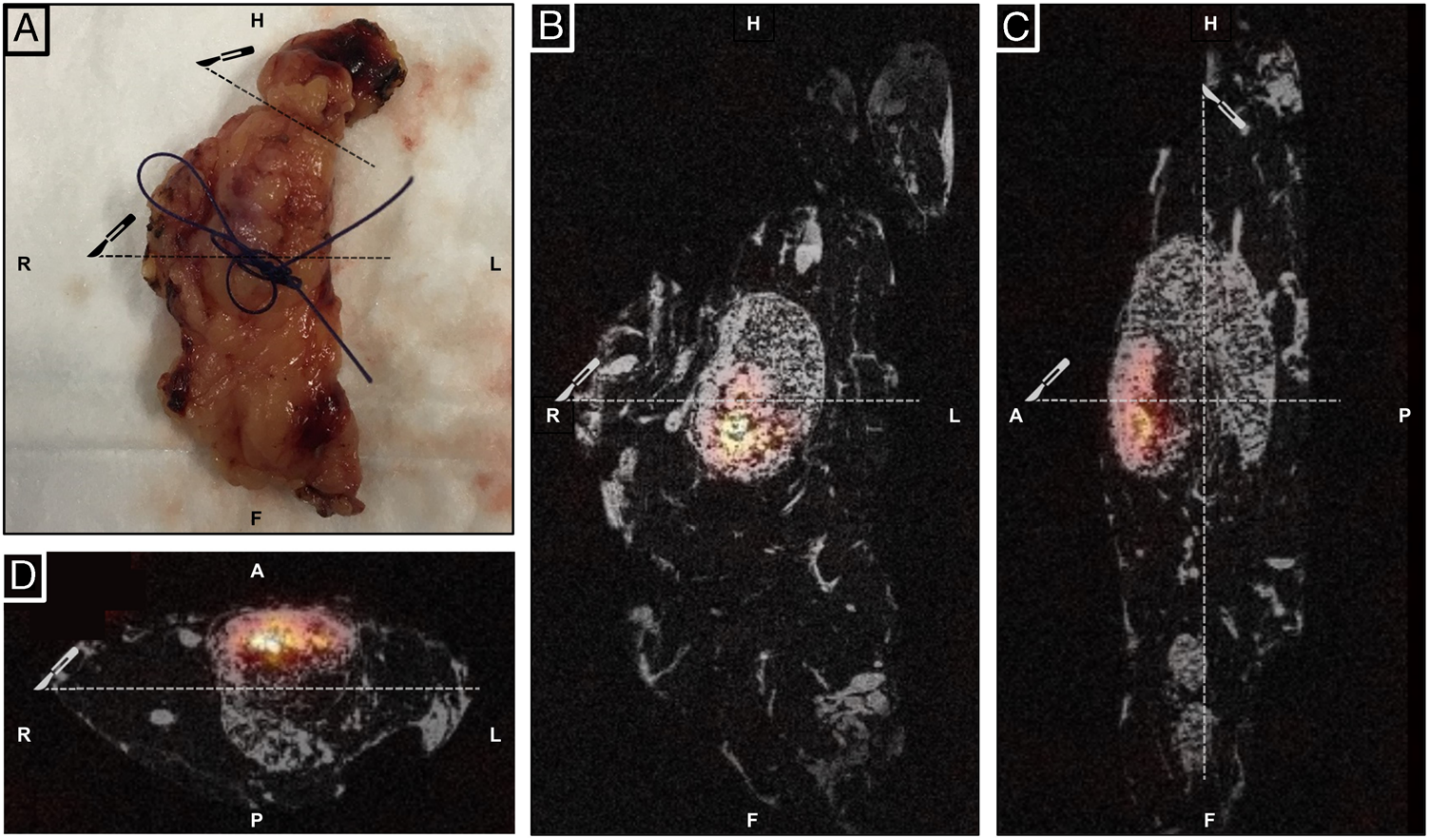
After surgery, ex vivo microSPECT imaging of LN packets containing suspicious lesions was performed (U-SPECT II, MILabs; 2.0 mm diameter pinhole collimator tube, 2 hours acquisition time). MRI was performed for anatomical reference using a 7T preclinical MR system (ClinScan; Bruker Biospin). **A**, Image shows a macroscopic image of the resected LN packet from the right obturator fossa. A suture marks the area with the increased radio signal (γ-probe). Fused SPECT and MRI scans of the LN packet show increased tracer uptake in a small part of the LN at the very anterior side in 3 directions (ie, coronal [**B**], sagittal [**C**], and transversal [**D**]). Subsequently, the surgical specimen was examined for the presence of LNs by palpation and stripped of nonlymphatic tissue as standard of care (dotted line at the top left part in **A**). The dotted lines (scalpel icon) represent the cutting lines of the histopathological tissue processing.

**FIGURE 3:**
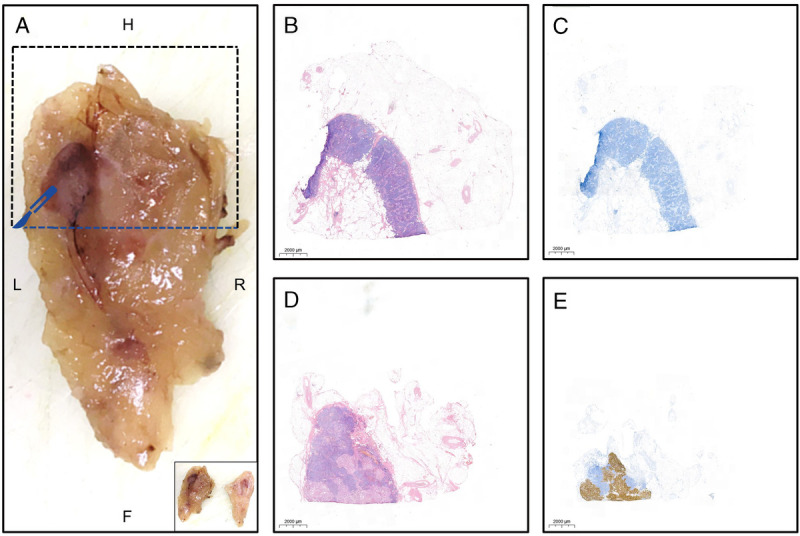
**A**, Image shows the macroscopic image of the anterior half of the surgical specimen from the obturator fossa (same as Figure 2A) after slicing it transversally in 2 parts, as illustrated in the image in the lower right corner of **A**. The photo shows the inner part of the anterior half of the sample, hence the alteration of left and right. Both halves (anterior and posterior) were subsequently cut in coronal direction as indicated by the dotted blue line for the anterior half, yielding 4 sections (or quarters) of the specimen, which were processed for paraffin embedding in separate cassettes (see also cutting lines in **C**). The paraffin blocks were cut at 3 μm slices and stained with hematoxylin-eosin and immunohistochemically for PSMA. Initially, none of the 4 cassettes showed a metastasis in the 9 × 12 × 8-mm LN. **B** and **C**, Images show the initial microscopic hematoxylin-eosin and PSMA-stained slides of the upper anterior section, respectively. However, based on intraoperative findings (γ-probe) and ex vivo imaging as described, suspicion of a metastasis persisted. The paraffin block of the respective (upper anterior) section was subsequently fully cut and reanalyzed, revealing a 7-mm (transversal) LN metastasis (**D**) with strong PSMA expression (**E**). This case illustrates that it is important to realize that histopathology, albeit the standard of reference, has its limitations due to sampling effects and could thus lead to false-negative results.^4–6^ To improve sensitivity of histopathology, molecular imaging can guide the pathologist by identifying suspicious LNs that potentially require additional and/or deeper cuts, before histopathological evaluation.^7^ This so-called image-guided pathology could both improve sensitivity and efficiency of the histopathological process and safeguard appropriate postoperative therapy for the patient.^8^
